# Perianal lidocaine application during unsedated colonoscopy: A double-blind randomized controlled trial

**DOI:** 10.1371/journal.pone.0348062

**Published:** 2026-04-30

**Authors:** Jiankun Wang, Yuwen Tao, Rui Wu, Lili Zhao, Li Liu, Zhining Fan, Wentao Fan

**Affiliations:** 1 Department of Geriatric Gastroenterology, the First Affiliated Hospital with Nanjing Medical University & Jiangsu Province Hospital, Nanjing, China; 2 Gastroenterology Department, the Fourth Affiliated Hospital of Nanjing Medical University, Nanjing, China; PRISM CRO, PAKISTAN

## Abstract

**Background and Aims:**

nsedated colonoscopy is often associated with patient discomfort and may increase procedural difficulty for endoscopists. This study aimed to investigate the influence of perianal lidocaine application on the comfort level of patients and endoscopic operations during unsedated colonoscopy.

**Methods:**

122 patients aged 18–40 years were enrolled and randomly allocated to colonoscopy with 2% lidocaine (experiment group, n = 61) or normal saline (control, n = 61) smeared around the anus. The primary outcomes were anal pain score and abdominal pain score during and after colonoscopy, assessed using a visual analogue scale (VAS). Secondary outcomes included cecal intubation time, polyp detection rate, adenoma detection rate, and adverse events.

**Results:**

There was no statistical difference between groups in terms of abdominal pain scores during unsedated colonoscopy. Abdominal pain scores after colonoscopy were significantly lower in the lidocaine group than in the saline group. (P < 0.001). However, the lidocaine group had significantly lower anal pain scores during and after colonoscopy (P < 0.001) and a shorter cecal intubation time (P < 0.001). Polyp and adenoma detection rates were higher in the lidocaine group (P < 0.05). Junior endoscopists achieved greater improvements in these outcomes compared to senior endoscopists.

**Conclusion:**

Perianal application of lidocaine may reduce anal pain and may improve procedural efficiency during unsedated colonoscopy, particularly among junior endoscopists.

## Introduction

Colonoscopy, the gold standard for colorectal cancer screening, has varying utilization rates across regions. For instance, in some developing countries, its adoption remains low due to resource limitations, while in the United States, the utilization is higher but limited by patient reluctance due to discomfort [1]. Many patients are reluctant to undergo colonoscopy, especially unsedated colonoscopy. Colonoscopy may cause both physical discomfort and psychological anxiety for patients. Many patients are afraid or even resistant to this operation, and this phenomenon leads to the failure of timely detection and treatment of many diseases. Although general anesthetized colonoscopy is becoming a common method. However, many primary hospitals still lack the resources to routinely perform sedated colonoscopy. During the unsedated colonoscopy, patients mainly composed of young adults will contract the anal sphincter significantly, which increases the friction between the colonoscopy and the anus due to tension and other factors, resulting in a difficult colonoscopy. On the one hand, the patient feels obvious pain, which leads to more tension and further contraction of the anal sphincter, forming a vicious cycle. On the other hand, the operation resistance increases, leading to difficulties in colonoscopy, increasing the rate of misdiagnosis and missed diagnosis.

Currently, Hyoscine butyl bromide and glucagon, which are anticonvulsants, are injected intravenously or intramuscularly during colonoscopy. But they can cause adverse events, including myosis, tachycardia, dry mouth, and hypoglycemia [2]. These drugs have not been applied universally, an ideal agent is needed to alleviate patients’ discomfort and improve the efficacy of colonoscopy. At the same time, recent studies have shown that local use of lidocaine in the colon can also inhibit spasticity, without obvious side effects [3]. All of the above are studies on the intestinal spasm. In the present study, we aimed to investigate the efficacy and safety of perianal lidocaine application on the comfort level of patients, and the influence of perianal lidocaine application on endoscopic operation during unsedated colonoscopy.

## Methods

### Study design/setting

We conducted a prospective, double-blind, randomized controlled trial to evaluate the effect of smear lidocaine around the anus before colonoscopy, compared with normal saline solution, at a tertiary-care hospital in China (Jiangsu Province Hospital). The study was approved by the Institutional Review Board (2020-SR-391) on **23/09/2020**. Written informed consent was obtained from all participants prior to enrollment. Participant data were collected and analyzed in a de-identified manner. Participant recruitment was conducted from **01/02/2021**–**01/01/2022,** and follow-up was completed by **08/01/2022**. This trial was registered at the **Chinese Clinical Trial Registry (ChiCTR)** with the registration number **ChiCTR2100042703**. All methods were performed in accordance with the relevant guidelines and regulations. The CONSORT (Consolidated Standards of Reporting Trials) guidelines were followed in reporting this study. The authors confirm that all ongoing and related trials for this intervention are registered.

### Participants

The inclusion criteria were as follows: (1) patients aged 18–40 years; (2) no perianal disease; (3) no previous history of colorectal and perianal surgery; (4) patients have no coagulation disorder, mental illness, and serious cardiopulmonary diseases. The exclusion criteria were as follows: (1) history of colorectal or perianal surgery; (2) suffering from inflammatory bowel disease and perianal disease; (3) allergic to lidocaine; (4) patients with severe organic diseases or coagulation disorders. The quality of bowel preparation was assessed according to the Boston Bowel Preparation Scale.

### Intervention and randomization

All subjects provided written informed consent before undergoing colonoscopy. Participants were randomly assigned (1:1) to either the lidocaine or saline group using a computer-generated random sequence prepared by an independent statistician. Allocation concealment was ensured by sequentially numbered, opaque, sealed envelopes prepared by a study coordinator not involved in patient recruitment or outcome assessment. Immediately before colonoscopy, a study nurse opened the next envelope and applied the allocated intervention. In the lidocaine group, patients underwent colonoscopy after evenly smearing 5 mL of 2% lidocaine gel (5 mL: 0.1 g; Hebei Tiancheng Pharmaceutical Company Limited) around the anus. In the control group, the same procedure was performed with 5 mL of normal saline. To maintain blinding, both interventions were prepared in identical syringes with indistinguishable appearance and volume. Patients and endoscopists were blinded to group allocation, and outcome assessors and data analysts remained masked until the completion of the primary analysis.

### Procedure

After undergoing a standard bowel preparation using polyethylene glycol electrolyte lavage solution, an unsedated colonoscopy was performed by both junior and senior endoscopists. Junior endoscopists were defined as residents or fellows with less than 3 years of clinical experience who had acquired sufficient skills to perform colonoscopy independently. Senior endoscopists, on the other hand, were defined as attending physicians with more than 5 years of clinical experience and a significantly higher procedural volume compared to junior endoscopists.

During the procedure, abdominal and anal pain scores were recorded at two time points: during and after the colonoscopy. The cecal intubation time, defined as the time taken to reach the cecum, and the polyp detection rate were also documented for each patient. In addition, adverse reactions observed during the examination were recorded, and all patients were followed up by telephone at 24 hours and 7 days post-procedure to monitor for any delayed complications. The structured follow-up included standardized questions regarding pain, discomfort, and any other symptoms experienced after the procedure.

### Outcome measures

The primary outcomes were anal pain score and abdominal pain score during and after colonoscopy. Patients were asked to rate the intensity of pain using a visual analogue scale (VAS) ranging from 0 to 10. The VAS was presented as a 10-cm horizontal line, with the left endpoint labeled ‘0’ (indicating no pain) and the right endpoint labeled ‘10’ (indicating the worst possible pain); patients indicated their perceived pain intensity by marking the corresponding point on the line (0–10 points). The secondary outcomes were cecal intubation time, polyp detection rate, adenoma detection rate, and adverse events. The cecal intubation time, defined as the time taken to reach the cecum, was recorded for all procedures to assess operator efficiency. The polyp detection rate is defined as the number of patients with at least one polyp divided by the number of screening colonoscopies. The adenoma detection rate is defined as the number of patients with at least one adenoma divided by the number of screening colonoscopies. The adverse events were observed during the examination and within 1 week after the examination. Follow-up was conducted via telephone within 24–48 hours after the procedure by trained research staff using a standardized questionnaire to record any delayed adverse events or patient-reported outcomes. According to the Boston Bowel Preparation Scale, Score<5 would likely be considered inadequate intestinal preparation.

### Sample size calculation

Based on a study that assumed a spasm inhibition rate of 78% with lidocaine and 50% with normal saline [3], we employed PASS 11 Power Analysis and Sample Size Software to detect a difference of at least 28% between the groups using the chi-squared test with a two-sided alpha error of 0.05 and a power (1-β) of 0.90. This required 60 patients in each group for the study. Our goal was to enroll 61 patients in each group.

### Statistical methods

All analyses were performed using SPSS version 31.0 (IBM Corp., Armonk, NY, USA). All variables were summarized descriptively: normally distributed continuous variables as mean ± standard deviation (SD), non-normally distributed continuous variables as median with interquartile range (IQR), and categorical variables as counts and percentages. For baseline comparisons, normally distributed continuous variables were analyzed with the independent-samples t-test, non-normally distributed continuous variables with the Mann–Whitney U test, and categorical variables with the Chi-square test when all expected cell counts were ≥5, or otherwise with Fisher’s exact test.

For analyses of primary and secondary outcomes (Tables 2 and 3), continuous outcomes were compared between groups using general linear regression models, with the treatment group entered as a binary independent variable (lidocaine group = 1, saline group = 0). Because several outcome variables were not normally distributed, bias-corrected and accelerated (BCa) bootstrap resampling with 1000 iterations was applied to obtain robust estimates of mean differences (MDs) with 95% confidence intervals (CIs). Although some variables were non-normal distributed, mean differences derived from regression models remain valid and clinically interpretable effect measures and were therefore reported instead of median differences. Categorical outcomes were analyzed using binary logistic regression models, and odds ratios (ORs) with 95% confidence intervals were reported as measures of effect size. All enrolled participants completed the assigned interventions and outcome assessments; therefore, no missing data occurred and no imputation procedures were required. Because the analyses were based on predefined primary and secondary outcomes, no adjustment for multiple comparisons was applied. All statistical tests were two-sided, and a P value <0.05 was considered statistically significant.

## Results

**Fig 1:**
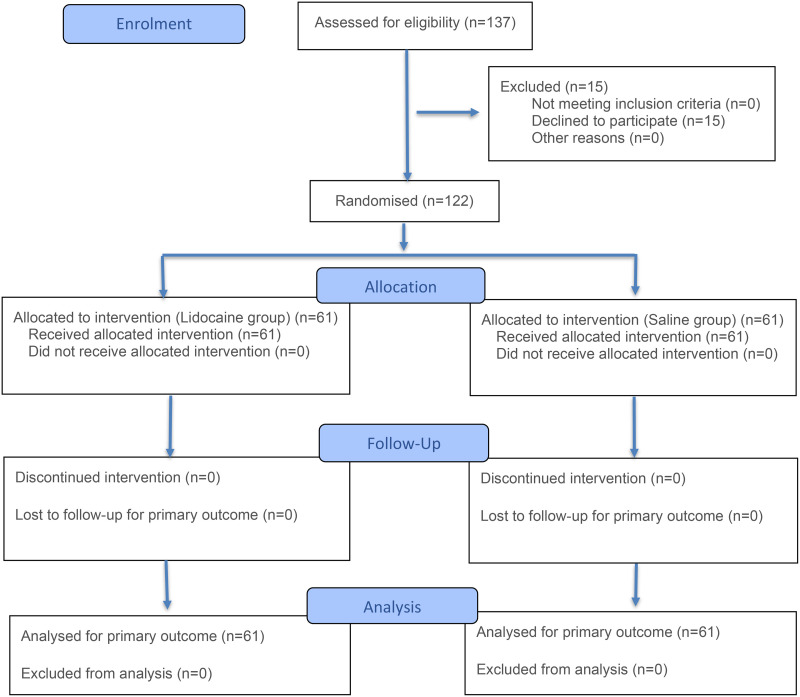
CONSORT flow diagram of participant enrollment, randomization, follow-up, and analysis.

From February 2021 to January 2022, 137 patients were eligible for inclusion and 15 patients declined to participate in the study. The remaining 122 patients were enrolled and were randomized to either the lidocaine group (n = 61) or saline group (n = 61) for colonoscopy (Fig 1). The 122 patients had a bowel preparation score≥5 and were eligible to undergo colonoscopy.

### Baseline characteristics

Patient ages were 30.31 ± 0.63 and 30.67 ± 0.61 in the lidocaine group and saline group, respectively. The male-to-female ratio was 43:18 in the lidocaine group and 42:19 in the saline group. Furthermore, the body mass index of the patients in the lidocaine (24.15 ± 0.42) was similar to that in the saline group (24.22 ± 0.41). The baseline characteristics of the patients mentioned previously had no significant differences between the lidocaine group and the saline group. The reasons patients underwent colonoscopy included bowel dysfunction (n = 46), Abdominal pain (n = 25), hematochezia (n = 20), and health examination (n = 11). There were no significant differences between the two groups in terms of the different causes of colonoscopy. Moreover, 17 patients in the lidocaine group and 18 patients in the saline group had previous abdominal surgery and no significant differences were found in previous operation history between the two groups. There was no significant difference in the bowel preparation score between the lidocaine group (score 7) and the saline group (score 6) (P = 0.538). There was no difference in the number of junior and senior endoscopists between the Lidocaine group and the Normal Saline group (Table 1).

**Table 1:** Baseline characteristics of the study participants in the lidocaine and saline groups.

	Total	Lidocaine group	Saline group	*P*-Value
	n = 122	n = 61	n = 61	
Age, years				
Mean ± SD, (range)	30.49 ± 0.44	30.31 ± 0.63	30.67 ± 0.61	0.682
	(20-40)	(20-40)	(22-40)	
Gender				
Female, n (%)	37 (30.33)	18 (29.51)	19 (31.15)	0.844
Male, n (%)	85 (69.67)	43 (70.49)	42 (68.85)	
Body mass index, kg/m^2^				
Mean ± SD, (range)	24.19 ± 0.29	24.15 ± 0.42	24.22 ± 0.41	0.907
	(17.97-31.56)	(18.33-31.56)	(17.98-31.15)	
The cause of colonoscopy				
bowel dysfunction, n (%)	46 (37.70)	22 (36.07)	24 (39.34)	0.975
Abdominal pain, n (%)	25 (20.49)	13 (21.31)	12 (19.67)	
hematochezia, n (%)	20 (16.39)	20 (32.79)	20 (32.79)	
health examination, n (%)	11 (9.02)	6 (9.84)	5 (8.20)	
Previous abdominal surgery				
Yes, n (%)	35 (28.69)	17 (27.87)	18 (29.51)	0.907
appendicectomy	17	7	10	
cholecystectomy	7	4	3	
cesarean	8	4	4	
hysteromyoma	3	2	1	
No, n (%)	87 (71.31)	44 (72.13)	43 (70.49)	
Bowel cleansing score				
P_50_, (P_25-75_)	6	7	6	0.538
	(6–8)	(6–8)	(6–8)	
Endoscopist				
junior endoscopist, n (%)	62 (50.82)	31 (50.82)	31 (50.82)	1
senior endoscopist, n (%)	60 (49.18)	30 (49.18)	30 (49.18)	

### Primary outcomes

All patients successfully underwent colonoscopy. Abdominal pain scores during colonoscopy did not differ significantly between the lidocaine and saline groups (p = 0.135) (Table 2). However, abdominal pain scores after colonoscopy were significantly lower in the lidocaine group than in the saline group (p < 0.001) (Table 2). Similarly, anal pain scores were significantly lower in the lidocaine group compared with the saline group, both during colonoscopy (p < 0.001) and after colonoscopy (p < 0.001).

**Table 2:** Comparison of primary and secondary outcomes between the lidocaine and saline groups.

	Total n = 122	Lidocaine group n = 61	Saline group n = 61	*P*-Value	Effect size (95% CI)
Cecal intubation time, minutes					
P_50_, (P_25-75_)	7.93	6.89	10.09	<0.001	MD = −2.99, BCa 95% CI −3.72 to −2.28
	(6.54-10.13)	(6.19-7.72)	(8.04-11.74)		
Polyp detection					
Yes, n (%)	30 (24.59)	22 (36.07)	8 (13.11)	0.002	OR = 4.01, 95% CI 1.68 to 9.60
No, n (%)	92 (75.41)	39 (63.93)	53 (86.89)		
Adenoma detection					
Yes, n (%)	16 (13.11)	12 (19.67)	4 (6.56)	0.040	OR = 3.49, 95% CI 1.06 to 11.52
No, n (%)	106 (86.89)	49 (80.33)	57 (93.44)		
Anal pain score during colonoscopy					
P_50_, (P_25-75_)	4	2	6	<0.001	MD = −3.36, BCa 95% CI −3.82 to −2.88
	(2–6)	(1–3)	(4–6)		
Anal pain score after colonoscopy					
P_50_, (P_25-75_)	3.5	2	5	<0.001	MD = −3.10, BCa 95% CI −3.43 to −2.77
	(2–5)	(2–3)	(5–6)		
Abdominal pain score during colonoscopy					
P_50_, (P_25-75_)	5	5	5	0.135	MD = −0.43, BCa 95% CI −0.97 to 0.14
	(4–7)	(4-6.5)	(5–7)		
Abdominal pain score after colonoscopy					
P_50_, (P_25-75_)	4	3	5	<0.001	MD = −1.75, BCa 95% CI −2.16 to −1.33
	(2.75-5)	(2–3)	(4–5)		
Adverse events					
Yes, n (%)	0	0	0		
No, n (%)	122	61	61		

Abbreviations: Continuous variables are presented as median (interquartile range), and categorical variables are presented as n (%).

MD, mean difference; OR, odds ratio; CI, confidence interval; BCa, bias-corrected and accelerated bootstrap.

### Secondary outcomes

The cecal intubation time was significantly shorter in the lidocaine group than in the saline group (p < 0.001). Moreover, the lidocaine group had significantly higher polyp detection rate (p = 0.002) and adenoma detection rate (p = 0.040) compared with the saline group. No adverse events were observed in either group (Table 2).

### Subgroup analysis

In the senior endoscopist subgroup, cecal intubation time was significantly shorter in the lidocaine group than in the saline group (p = 0.002) (Table 3). For the polyp detection rate, no statistically significant difference was observed between the lidocaine and saline groups (p = 0.096). Similarly, adenoma detection rate did not differ significantly between the two groups (Table 3). Abdominal pain scores during colonoscopy did not differ significantly between the two groups (p = 0.065) (Table 3). However, postoperative abdominal pain scores were significantly lower in the lidocaine group (p < 0.001). Anal pain scores were also significantly lower in the lidocaine group both during colonoscopy (p < 0.001) and after colonoscopy, (p < 0.001) (Table 3). These findings suggest that the application of lidocaine gel may be particularly beneficial for junior endoscopists.

**Table 3:** Subgroup analysis of primary and secondary outcomes according to endoscopist experience.

	Junior endoscopist		*P*-Value	Effect size (95% CI)	Senior endoscopist		*P-Value*	Effect size (95% CI)
	Lidocaine group	Saline group			Lidocaine group	Saline group		
	n = 31	n = 31			n = 30	n = 30		
Cecal intubation time, minutes								
P_50_, (P_25-75_)	7.20	10.58	<0.001	MD = −3.84, BCa 95% CI −4.75 to −2.93	6.52	8.46	0.002	MD = −2.11, BCa 95% CI −3.30 to −1.09
	(6.38-7.92)	(9.69-12.39)			(5.99-7.26)	(6.53-10.63)		
Polyp detection								
Yes, n (%)	10 (32.26)	2 (6.45)	0.007	OR = 6.74, 95% CI 1.68 to 27.01	12 (40.00)	6 (20.00)	0.096	OR = 2.67, 95% CI 0.84 to 8.46
No, n (%)	21 (67.74)	29 (93.55)			18 (60.00)	24 (80.00)		
Adenoma detection								
Yes, n (%)	4 (16.67)	1 (3.23)	0.194	OR = 4.44, 95% CI 0.47 to 42.26	8 (26.67)	3 (10.00)	0.107	OR = 3.27, 95% CI 0.77 to 13.83
No, n (%)	26 (83.33)	30 (96.77)			22 (73.33)	27 (90.00)		
Anal pain score during colonoscopy								
P_50_, (P_25-75_)	2	6	<0.001	MD = −3.90, BCa 95% CI −4.50 to −3.36	2	5	<0.001	MD = −2.80, BCa 95% CI −3.32 to −2.24
	(1–3)	(5–7)			(1–2)	(4–6)		
Anal pain score after colonoscopy								
P_50_, (P_25-75_)	2	5	<0.001	MD = −3.32, BCa 95% CI −3.85 to −2.74	2	5	<0.001	MD = −2.87, BCa 95% CI −3.30 to −2.44
	(1–3)	(5–6)			(2–3)	(4–6)		
Abdominal pain score during colonoscopy								
P_50_, (P_25-75_)	6	5	0.693	MD = −0.16, BCa 95% CI −1.00 to −0.65	5	5.5	0.065	MD = −0.70, BCa 95% CI −1.43 to 0.03
	(4–7)	(5–7)			(4-6.25)	(5-7.25)		
Abdominal pain score after colonoscopy								
P_50_, (P_25-75_)	3	5	<0.001	MD = −1.90, BCa 95% CI −2.48 to −1.31	3	4.5	<0.001	MD = −1.60, BCa 95% CI −2.20 to −1.00
	(2–3)	(4–5)			(2-3.25)	(4–5)		
Adverse events								
Yes, n (%)	0	0			0	0		
No, n (%)	31	31			30	30		

Abbreviations: Continuous variables are presented as median (interquartile range), and categorical variables are presented as n (%). MD, mean difference; OR, odds ratio; CI, confidence interval; BCa, bias-corrected and accelerated bootstrap.

## Discussion

Colonoscopy is an effective diagnostic method for colorectal diseases, but patient acceptance remains limited because of procedure-related discomfort. especially young people. The painful and unpleasant experience during colonoscopy results in poor tolerance to colonoscopy. Young people are more likely to suffer from tension and are more sensitive to pain. Various adverse events and complications can reduce their willingness to undergo a colonoscopy even when it is necessary [4–6]. What is more, physical and psychological discomfort makes patients cooperate with endoscopists badly. And this will weaken the efficiency of colonoscopy. Many methods have been tried to alleviate the discomfort of patients and improve the efficiency of colonoscopy. The most common way is a general anesthetized colonoscopy. However, an anesthetized colonoscopy costs more money and needs higher skill requirements. Therefore, anesthetized colonoscopy has been unable to replace unsedated colonoscopy. Antispasmodic agents are used to relax the muscle tone in the gastrointestinal tract during the colonoscopy, such as Hyoscine butylbromide and glucagon [2,7–10]. However, their adverse events have made the use of these two drugs controversial, such as dry mouth, miosis, urinary retention, palpitations (for hyoscine butylbromide), and reactive hypoglycemia and hyperglycemia (for glucagon) [2,11]. Warm water infusion can moderately reduce the patient’s discomfort, but does not suppress intestinal spasms [12–14]. The inhibitory effect of peppermint oil solution can prevent intestinal spasms with the side effects of short duration and recurrent spasms [15]. In addition, many methods have been successfully used for reducing pain during colonoscopy, including a small-diameter extra-flexible colonoscope [16], using a computer-based endoscopy simulator to train physician [17], listening to music during the procedure [18–20], use of magnetic endoscope imaging to avoid loop formation and colon stretching [21], and carbon dioxide insufflation [22].

Lidocaine is a local anesthetic and anti-arrhythmic drug commonly used in clinical diagnosis and treatment. It is mainly used in local infiltration anesthesia, epidural anesthesia, surface anesthesia, and nerve conduction block. It belongs to the amide class and has a long anesthetic effect time, good anesthetic effect, strong penetration, high safety, and simple administration. Due to its extremely low incidence of adverse reactions in diagnosis and treatment, lidocaine is commonly used in all kinds of simple and minor operations and is known as “all-purpose anesthetics” [23]. It is still controversial whether lidocaine is useful for colonoscopy. Nemoto et al proved that perianal lidocaine application was an effective and safe method for suppressing colorectal spasms during unsedated colonoscopy [3]. On the contrary, Cengiz et al showed that the use of perianal local anesthesia did not improve patients’ discomfort during unsedated colonoscopy [24]. This difference was related to the selection of patients and evaluation index. In the present trial, we recruited patients aged 18–40 years. Because young people are more likely to contract the anal sphincter significantly during unsedated colonoscopy, which leads to colonoscopy difficult. A long operation will increase abdominal and anal discomfort and decrease the efficiency of the examination. The anal sphincter of the elderly is weak with no obvious effect, so the treatment crowd of local anesthesia is mainly young adults. And to prevent unexpected adverse events, the old aged were excluded. Our findings suggest that perianal lidocaine application may be associated with lower anal pain scores during unsedated colonoscopy, which can be attributed to the anesthetic impact of lidocaine on sensory nerves within the mucosal layer [3]. Furthermore, patient education regarding the application of anesthesia to the colonoscope before intubation serves to provide psychological comfort and reassurance. This approach typically results in the relaxation of the majority of patients, fostering their cooperation during the procedure. By diverting their attention and gaining their trust, this technique aids in mitigating anxiety and reducing the excitability of the vagus nerve. This may contribute to improved patient tolerance during the procedure, although the underlying mechanism was not directly examined in this study.

The application of lidocaine gel in colonoscopy effectively reduces patient discomfort, leading to an enhanced success rate of the procedure. Adenoma detection rate (ADR) serves as a recognized quality indicator for colonoscopy, and several studies have suggested that the polyp detection rate (PDR) can be a reliable surrogate for ADR [25]. Multiple factors have been identified as influencing ADR or PDR, including the administration of midazolam/fentanyl during colonoscopy and the proficiency of the colonoscopist [26]. Consequently, in the lidocaine group, the procedure duration was shorter, and the polyp detection rate was higher. Although local anesthesia couldn’t alleviate abdominal pain during colonoscopy, which is associated with the operator’s experience and skill, it did provide relief from abdominal pain after the procedure. That may be because the shorter cecal intubation time could reduce excessive air filling into the intestinal lumen, which relieved abdominal discomfort. Furthermore, the perianal application of Lidocaine proved to be more beneficial for junior endoscopists compared to senior endoscopists.

It enables junior endoscopists to significantly reduce the cecal intubation time during endoscopic procedures, alleviate patients’ anal discomfort, and increase the detection rate of polyps. One possible explanation is that reduced patient discomfort may improve cooperation during colonoscopy, which could be particularly helpful for less experienced endoscopists. However, this interpretation remains speculative and warrants further study. No relevant adverse events were found in the lidocaine group. Perianal application of lidocaine was proved to be a safe method for colonoscopy. Some limitations of our study are acknowledged. First, the study was performed in one single medical center, the results of our study need to be further verified in a multicenter, large-scale trial. Second, for security and effectiveness reasons, we did not include any patients who were older than 40. It may cause selection bias. Third, although randomization reduced potential confounding, the sample size was relatively modest, particularly for subgroup analyses. Fourth, this study focused on clinical outcomes and did not directly investigate the mechanisms underlying the observed associations. In conclusion, the present study is one of the few on this subject and suggests that perianal lidocaine application may improve patient comfort and procedural efficiency during unsedated colonoscopy.

### What does this paper add to the literature?

This paper demonstrated that the perianal application of lidocaine was a safe and beneficial method for unsedated colonoscopy, resulting in alleviating patients’ discomfort and improving the efficiency of colonoscopy.

## Supporting information

S1 File Minimal dataset. (XLSX)
